# Intradetrusorial Botulinum Toxin in Patients with Multiple Sclerosis: A Neurophysiological Study

**DOI:** 10.3390/toxins7093424

**Published:** 2015-08-26

**Authors:** Antonella Conte, Antonella Giannantoni, Marilena Gubbiotti, Simona Pontecorvo, Enrico Millefiorini, Ada Francia, Massimo Porena, Alfredo Berardelli

**Affiliations:** 1 Department of Neurology and Psychiatry, University of Rome Sapienza, Rome 00185, Italy; E-Mails: antonella.conte@uniroma1.it (A.C.); enrico.millefiorini@uniroma1.it (E.M.); ada.francia@uniroma1.it (A.F.); 2 IRCCS Neuromed, Pozzilli, Isernia 86077, Italy; E-Mail: simonapontecorvo@yahoo.it; 3 Department of Surgical and Biomedical Sciences, Urology and Andrology Clinic, University of Perugia, Perugia 06156, Italy; E-Mails: agianton@libero.it (A.G.); marilena.gubbiotti@gmail.com (M.G.); massimo.porena@unipg.it (M.P.)

**Keywords:** multiple sclerosis, bladder dysfunction, H reflex, botulinum toxin, viscerosomatic reflex

## Abstract

Patients with multiple sclerosis (MS) often complain of urinary disturbances characterized by overactive bladder syndrome and difficulties in bladder emptying. The aim of the study was to investigate the pathophysiology of bladder dysfunction and the neurophysiological effects of intradetrusorial incobotulinum toxin A (BoNT/A) in patients with MS having both brain and spinal MS-related lesions. Twenty-five MS patients with neurogenic detrusor overactivity (NDO) underwent clinical evaluation and soleus Hoffmann reflex (H reflex) study during urodynamics. Of the 25 patients, 14 underwent a further session one month after intradetrusorial BoNT/A injection. Eighteen healthy subjects acted as the control. In healthy subjects, the H reflex size significantly decreased at maximum cystometric capacity (MCC), whereas in MS patients with NDO, the H reflex remained unchanged. In the patients who received intradetrusorial BoNT/A, clinical and urodynamic investigations showed that NDO improved significantly. Volumes at the first, normal and strong desire to void and MCC increased significantly. Despite its efficacy in improving bladder symptoms and in increasing volumes for first desire, normal and strong desire to void, BoNT/A left the H reflex modulation during bladder filling unchanged. In the MS patients we studied having both brain and spinal MS-related lesions, the H reflex size remained unchanged at maximum bladder filling. Since this neurophysiological pattern has been previously found in patients with spinal cord injury, we suggest that bladder dysfunction arises from the MS-related spinal lesions. BoNT/A improves bladder dysfunction by changing bladder afferent input, as shown by urodynamic findings on bladder filling sensations, but its effects on H reflex modulation remain undetectable.

## 1. Introduction

Bladder dysfunction in patients with multiple sclerosis (MS) often causes urinary symptoms characterized by overactive bladder syndrome (OAB) and difficulties in bladder emptying. Bladder dysfunction investigated with urodynamic tests includes detrusor overactivity (DO) with or without detrusor-sphincter dyssynergia [1,2,3].

Neurophysiological techniques can provide insights into the pathophysiology of bladder dysfunction in neurological conditions. In healthy subjects, previous studies showed that bladder filling inhibits soleus Hoffmann reflex (H reflex) size, and the decrease is thought to be due to the effect of bladder afferents input on spinal motor neurons [4]. In patients with neurogenic bladder, changes in the H reflex size depend on whether the central nervous system lesion lies in a suprapontine or spinal site. In patients with suprapontine neurological conditions, including vascular encephalopathy and Parkinson’s disease (PD), H reflex size decreases to a lesser extent than normal subjects, whereas in patients with spinal cord injury, it remains almost unchanged or it increases [5,6]. No published studies have investigated changes in the H reflex size during urodynamic assessment in patients with MS. H reflex testing may clarify whether bladder symptoms in MS depend on suprapontine or spinal lesions. Having this information may give further insight into the pathophysiology of bladder symptoms in MS patients.

Numerous studies show that patients with drug-refractory OAB symptoms related to neurological conditions, including MS, respond well to therapy with intradetrusorial botulinum toxin A injection (BoNT/A) [7,8,9,10,11,12,13]. In these studies, the investigators assessed improvement in urinary symptoms and bladder dysfunction with urodynamic investigation and quality-of-life (QOL) questionnaires. Some evidence in patients with neurogenic detrusor overactivity (NDO) related to spinal cord lesions or PD shows that BoNT/A injection improves bladder function by acting on the bladder afferents, as well as on the efferent pathways [6,11]. If BoNT/A modulates bladder afferent input, then we would expect that toxin injection influences H reflex modulation during bladder filling, but no study has yet investigated whether in patients with MS, BoNT/A improves bladder dysfunction by inducing changes in the H reflex size during bladder filling.

The first aim of the study was to investigate whether in MS, bladder dysfunction depends on suprapontine or spinal lesions. To do so, in patients with MS, we sought whether soleus H reflex during urodynamics decreases in size, therefore suggesting a “suprapontine mechanism” for bladder dysfunction, or remains unchanged, therefore suggesting a “spinal mechanism” responsible for bladder dysfunction. The second aim of this study was to investigate whether, in these patients, intradetrusorial BoNT/A injection induces changes in the H reflex modulation during bladder filling. To do so we therefore tested soleus H reflex during bladder filling before and one month after a single intradetrusorial BoNT/A injection.

## 2. Experimental Section

### 2.1. Patients

We enrolled 25 patients (aged: 44 ± 2 years) with MS and NDO and OAB symptoms and 18 healthy subjects (aged: 39 ± 3 years) attending the Department of Urology and Andrology at the University of Perugia and the Department of Neurology and Psychiatry, “Sapienza”, University Rome. The experimental procedures were carried out in accordance with the Declaration of Helsinki and approved by the institutional review board of the University of Perugia. All participants gave their informed written consent. All patients had a diagnosis of MS according to modified McDonald criteria [14]. All patients therefore had magnetic resonance imaging (MRI) scans showing periventricular, juxtacortical, infratentorial and spinal lesions. Table 1 shows their demographic features and MRI findings on spinal lesion localizations. All of the patients studied had OAB and NDO symptoms refractory to conventional anticholinergic therapy (at least three antimuscarinic agents—tolterodine, oxybutynin and solifenacin—each taken for at least 1 month). In all patients, oral anticholinergics were discontinued 1 month before entry into the study. Patients maintained skeletal muscle antispastic therapy unchanged throughout the study. Exclusion criteria were OAB symptoms due to bladder outlet obstruction, recurrent urinary tract infections, cognitive impairment, pregnancy, anticoagulant therapy, psychoactive agents modulating bladder function (venlafaxine, amitriptyline), aminoglycosides and other drugs thought to interfere with bladder function and patients with radiculopathy and peripheral neuropathy. Patients were also informed about the possible need for intermittent catheterization after BoNT/A treatment. Preliminary urologic assessment included history taking, physical examination, serum laboratory tests, urinalyses and culture and upper and lower urinary tract ultrasound. Daytime and nighttime urinary frequency and episodes of urinary incontinence were recorded in a 3-day voiding diary. Patients also completed a standardized QoL questionnaire on incontinence (I-QoL) [15]. Patients indicated to what extent bladder problems limited their daily life activities on a visual analog scale (VAS) consisting of a 10-cm line marked with “not at all” at the right end and “very much” at the left end. Visual ratings (“not at all” to “very much”) were then converted by the investigators to numerical values using a 0–10 scale [16].

### 2.2. Urodynamic Assessment

All MS patients underwent urodynamic assessment including electromyographic recording from the external urethral sphincter muscle [3]. During cystometry, volumes at first, normal and strong desire to void and first volume and maximum pressure of uninhibited detrusor contractions (UDC: first volume and UDC-pmax) and maximum cystometric capacity (MCC) were recorded. In all patients, we also recorded the detrusor pressure at MCC (pDetMCC) and post-void residual volume (PVR).

**Table 1:** Clinical and demographic features of patients with multiple sclerosis.

Patients	Sex (M/F)	Age (Years)	Disease Duration (Years)	MS Subtypes	EDSS	MRI Spinal Lesion Localization
1	F	44	15	RR	2.5	C4-C6
2	F	41	15	RR	3.5	C5-C7
3	F	57	38	SP	6.5	C4-C5, D6-D9
4	F	56	16	RR	3.0	C3-C4, C4-C5, C5-C6, C7
5	M	45	18	RR	3.5	C4, D6-D7
6	F	37	14	RR	3.0	C3-C5
7	F	39	21	RR	4.0	C4-C6, D5-D6
8	F	51	29	RR	4.0	C3-C4, D3, D6
9	F	36	14	RR	2.5	C7-D1, D6, D7
10	F	47	15	SP	6.0	C2, C5, C7
11	F	46	24	RR	2.5	C3, D2-D3
12	M	54	38	RR	5.0	C2-C3, C6, D4
13	M	43	5	RR	2.5	C2, D4, D7, D9
14	M	33	10	RR	4.0	C2-C3, C4-C5, C6, D3
15	F	57	16	RR	2.5	C5
16	F	47	20	RR	6.0	C3, C6, D2-D4
17	M	58	31	SP	6.5	C2-C3, D6-D8
18	F	26	7	RR	3.0	C4-C5, D3, D4
19	F	49	14	SP	6.0	C4-C6
20	M	27	9	RR	1.0	D9-L1
21	F	32	14	RR	1.0	C4, D2, D6
22	F	54	17	RR	4.5	C2-C3, C6-C7, D6, D7
23	F	35	7	RR	2.5	C3, C6, D4
24	F	60	30	RR	4.0	C1-C2, D5, D9
25	F	30	8	RR	2.5	C5, C7, D6
Mean ± SE	6M, 19F	44.1 ± 2	17.8 ± 2	-	3.7 ± 0.3	-

EDSS: Kurtzke’s Expanded Disability Status Scale; MRI: magnetic resonance imaging; MS multiple sclerosis; RR relapsing-remitting; SP secondary progressive.

### 2.3. Neurophysiological Evaluation

Patients underwent the H reflex study at the same time as urodynamic assessment. Because evidence in humans suggests that hip angle is critical for soleus H reflex modulation [17], during H reflex recordings and urodynamic assessment before and after BoNT/A injection, subjects lay supine in a gynecological position (needed for urodynamic assessment) and kept the hip angle unchanged during the experimental procedures (bladder empty and full bladder assessment, before and post-BoNT/A injection assessments).

Electrical stimuli were delivered to the right tibial nerve with an electromyographic (EMG) device (Myohandy matrix line, Micromed SpA, Mogliano Veneto, TV, Italy) through a monopolar needle electrode placed in the popliteal fossa and the anode at the knee cap. The stimulating electrode delivered percutaneous electrical stimuli of 1 ms square-wave pulses. At empty bladder, we increased the stimulation intensity in 1-mA steps, in order to obtain a maximum H reflex with the lowest M wave amplitude. The measurement of the maximum H reflex was taken when a further increase in stimulation intensity determined a decrease in the H reflex size. The intensity was then adjusted to obtain an H reflex size that was about 50% of the maximum H reflex and a low amplitude M wave (stimulation intensity was therefore lower than that for maximum H reflex size). To check the efficacy and stability of tibial nerve stimulation, the M wave size was measured at the beginning of the H reflex recording and monitored throughout the testing. Stimulation intensity for the H reflex testing at maximum bladder filling was adjusted to obtain an M wave with the same size recorded at empty bladder. In patients with MS, who received intradetrusor BoNT/A injection, stimulation intensity was adjusted to obtain an M wave from the soleus muscle with the same size of that recorded during the session before BoNT/A injection. With this precaution, we confidently exclude that any changes in the H reflex size during the experimental procedure performed after BoNT/A injection did not depend on changes in the amount of tibial nerve fibers activated by the electrical stimuli. EMG signals were recorded from the soleus muscle (bandwidth: 20 Hz–1 kHz) by using Ag/AgCl surface recording electrodes in a belly-to-tendon configuration. In each patient, we collected 10 H reflexes, repeated at 10-s intervals, for each condition. H reflex and M wave amplitudes were measured peak-to-peak. Mean values were then calculated for each condition in every single patient and entered in the data analysis.

The H reflex was tested under two bladder filling conditions: empty bladder (control value), maximum bladder capacity (when subjects feel they can no longer delay micturition).

The empty bladder condition always preceded full bladder assessment.

### 2.4. Botulinum Toxin Injection

After the baseline urodynamic and neurophysiological assessment, patients underwent intradetrusor injections of incobotulinum toxin A (Xeomin^®^, Merz Pharmaceuticals, Frankfurt, Germany), a commercially-available type A serotype, according to the technique originally described elsewhere [3,9,10] (Proietti, *et al.*, Clinical and urodynamic effect of Xeomin. European Journal of Neurology 2011; 18. 15° Congress of European Federation of Neurological Societies). Of the 25 patients with MS, 14 received a single BoNT/A toxin injection, 100 U diluted in 10 mL NaCl into the detrusor muscle at 10 sites, including the trigone, under cystoscopic control. All procedures were performed under short-lasting fentanyl general anesthesia on an inpatient basis. Immediately after BoNT/A injections, a 16 Ch Foley indwelling catheter was inserted for 24 h. The catheter was then removed, and patients were discharged only after checking whether they spontaneously voided and did not have hematuria.

### 2.5. Experimental Design

All patients enrolled underwent clinical, I-QoL, voiding diary, urodynamics, ultrasound and neurophysiologic assessment at baseline. Of these 25 patients, 14 underwent the whole experimental procedure in a further session 1 month (T1) after BoNT/A injection. Data on changes in the H reflex size at maximum bladder filling were compared to those in a group of 18 healthy subjects.

### 2.6. Statistical Analysis

SPSS software (SPSS Inc., Chicago, IL, USA) was used for statistical analysis. The non-parametric Wilcoxon’s test was used to evaluate changes in I-QoL, VAS, daytime and nighttime urinary frequency and changes in the urodynamic variables. To evaluate changes in the H reflex size, having excluded any assumption violation for ANOVA (Kolmogorov-Smirnov test, Box’s test, Levene’s test), between group repeated-measure analysis of variance (ANOVA) was used to compare changes in the H reflex size during urodynamic assessment in patients with MS *vs.* healthy subjects. We also ran a between group ANOVA to compare changes in the H reflex size during bladder filling between MS patients with and without dyssynergic EMG activity during urodynamics. Repeated-measure ANOVA was also used to evaluate changes in the H reflex size in patients with MS before and one month after BoNT/A injection. The *t*-test was used for *post hoc* analysis. To investigate possible correlations between the clinical, urodynamic and neurophysiologic variables, we used post-BoNT/A/pre-BoNT/A values for each variable and determined Spearman’s correlation coefficient. Holmes’ correction for multiple comparisons was applied. All values are expressed as the mean ± SE. *p* < 0.05 was considered to indicate statistical significance.

## 3. Results

None of the patients complained of systemic adverse events during or after the BoNT/A treatment. None of the patients enrolled declared that the experimental procedures were painful nor retired their consent to the study during the experimental procedures due to pain/discomfort. During the one month follow-up, no urinary tract infections were reported.

### 3.1. Clinical and Urodynamic Findings

At baseline, all patients studied complained of increased daytime and nighttime urinary frequencies (episodes/day: 7.3 ± 0.9; episodes per night: 2.8 ± 0.5). They also complained of urgency and had low I-QoL scores (57.5 ± 4) and VAS scores (4.3 ± 0.2). In patients with MS, urodynamic assessment detected detrusor overactivity: UDC-first volume, 161 ± 45 mL; UDC pmax, 33 ± 12 cm H_2_O) and MCC 267 ± 39 mL. All patients spontaneously emptied their bladder with a low post-void residual volume (PVR: 56 ± 18 mL). Fifteen out of twenty-five patients with MS showed dyssynergic EMG activity during urodynamics. Conversely, urodynamic assessment in healthy subjects showed no uninhibited detrusor contractions, MCC 460 ± 24 mL and no PVR in any subject.

After BoNT/A injection, in patients with MS, Wilcoxon’s test showed that daytime and nighttime urinary frequency, as well as daily urinary incontinence frequency diminished significantly (daytime urinary frequency: 8.5 ± 1.2 pre-BoNT/A *vs.* 5.2 ± 0.3 post-BoNT/A, *p* = 0.02; nighttime urinary frequency 2.42 ± 0.4 pre-BoNT/A *vs.* 0.5 ± 0.2 post-BoNT/A, *p* < 0.001). Wilcoxon’s test also showed a significant improvement in VAS scores (*p* = 0.0002) and in I-QoL (*p* = 0.01).

On urodynamics after BoNT/A treatment, volumes at first, normal and strong desire to void and MCC significantly increased (first desire: *p* = 0.01; normal desire: *p* = 0.01; strong desire: 0.02; MCC: *p* = 0.03). Twelve out of the 14 patients who received BoNT/A injection showed no dyssynergic activity during urodynamics at the post-BoNT/A assessment. PVR, pDetQmax and Qmax remained unchanged (PVR: *p* = 0.15; pDetQmax: *p* = 0.67; Qmax: *p* = 0.43).

### 3.2. Neurophysiologic Assessment

**Figure 1:**
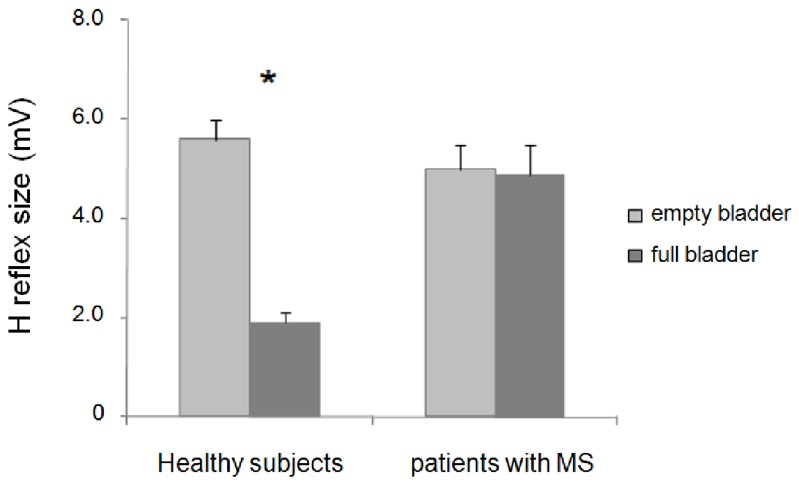
Changes in H reflex size during bladder filling in healthy subjects and patients with multiple sclerosis (MS); * indicates statistical significance.

Between group repeated-measures ANOVA for changes in the H reflex size during bladder filling at the baseline assessment in patients with MS and healthy subjects showed a significant effect of factor bladder filling (F_1,41_ = 27.67; *p* < 0.0001), a significant two-way interaction between factors bladder filling and group (F_1,41_ = 31.71; *p* < 0.0001) and a non-significant factor group (F_1,41_ = 3.97; *p* = 0.05). *Post hoc* analysis showed that in healthy subjects, the H reflex size was significantly inhibited at MCC (*p* < 0.001), whereas in patients with MS, it remained almost unchanged (*p* = 0.71) (Figure 1; Table 2). Unlike the H reflex, the M wave remained unchanged throughout the experiments (bladder filling: F_1,41_ = 0.11; *p* = 0.73; bladder filling and group interaction: F_1,41_ = 1.57; *p* = 0.22) (Table 2).

**Table 2:** Neurophysiological findings in healthy subjects and patients with multiple sclerosis (MS) at maximum cystometric capacity (MCC), BoNT/A, botulinum toxin A.

Group	H Reflex Size at MCC (% H Reflex at Empty Bladder)	M Wave Size at MCC (% M Wave at Empty Bladder)
Healthy subjects	34.4 ± 3.9	102.3 ± 1.3
MS patients (25)	97.8 ± 8.9	105.2 ± 2.2
MS patients pre BoNT/A (14)	88.4 ± 8.3	105.4 ± 2.0
MS patients post BoNT/A (14)	97.4 ± 5.9	99.3 ± 2.4

Between-group ANOVA to compare percentage changes in H reflex size during bladder filling between MS patients who had dyssynergic activity and those who did not showed no significant differences between the two groups (F_1,24_ = 0.01; *p* = 0.89).

**Figure 2:**
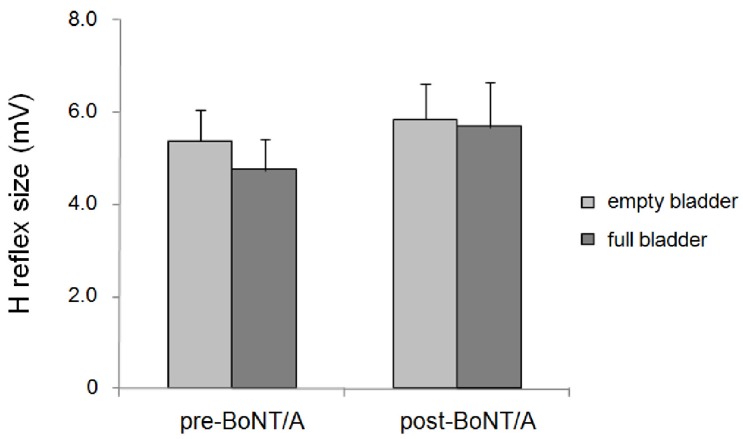
Changes in H reflex size in patients with multiple sclerosis during bladder filling before and after incobotulinum toxin-A (BoNT/A) injection.

ANOVA for changes in the H reflex size during bladder filling before and after BoNT/A injection in patients with MS showed no significant effect of factor BoNT/A (F_1,13_ = 1.61; *p* = 0.22), bladder filling (F_1,13_ = 2.78; *p* = 0.11) or interaction between BoNT/A and bladder filling (F_1,13_ = 0.39; *p* = 0.54) (Figure 2; Table 2). The stimulation intensity needed to obtain 50% of the maximum H reflex did not significantly vary across the two sessions (*p* = 0.3).

### 3.3. Correlation between Urodynamic and Neurophysiologic Variables

No significant correlations were found between clinical, urodynamic and neurophysiologic variables.

## 4. Discussion

The new finding in this study is that whereas in healthy subjects, the H reflex size at MCC decreases, in MS patients with detrusor overactivity, H reflex size remains unchanged. In the patients who received BoNT/A injection, clinical and urodynamic investigations showed a significant improvement in drug-refractory OAB symptoms. Despite its efficacy in improving bladder symptoms and in increasing volumes for first, normal and strong desire during urodynamics, we now show that BoNT/A had no significant effect on the H reflex.

To ensure reliable data, our experimental procedures envisaged several precautions. For example, to exclude changes in the hip joint angles [17] that might have influenced the H reflex modulation during bladder filling, all participants maintained the same position throughout the experimental procedure. To circumvent possible H reflex changes due to differences in the number of peripheral axons activated by electrical stimuli, we constantly monitored the M wave amplitude and kept it unchanged during the experimental session. In the patients who received BoNT/A injection, we chose the intensity for the H reflex study at the post-injection assessment to obtain an M wave size equal to that before the injection. The observation that M wave and H reflex size at empty bladder before and one month after intradetrusorial BoNT/A injection remained unchanged demonstrates that no significant changes in the tibial nerve stimulation in the two sessions occurred. Equally important, all patients had their urine tested before each session to exclude urinary tract infections that might influence urodynamic and neurophysiologic data.

All confounding excluded, our study gives insight into the pathophysiology underlying bladder dysfunction in patients with MS showing that, unlike healthy subjects, in patients with MS, who had MS-related lesions in the brain and in the spinal cord, bladder filling fails to modulate the H reflex size. These findings match those obtained in previous studies from our group and an earlier study by others in patients with spinal cord injury showing that H reflex size at maximum bladder filling remains statistically unchanged or slightly increases in size [6,18]. In patients with complete spinal cord transection, the pontine reticular formation loses its control over the bladder, therefore interrupting somatic motoneuron inhibition, resulting in sphincteric dyssynergia. In patients with suprapontine lesions (multi-infarct encephalopathy, Parkinson’s disease), maximum bladder filling decreases the H reflex size [5,6]. The MS patients we studied all had lesions in the brain, as well as in the spinal cord; thus, lesion load in the brain and in the spinal cord might have contributed to the OAB symptoms. Since in the MS patients we studied, the H reflex remained statistically unchanged at maximum bladder filling and this neurophysiological pattern has been previously found in patients with spinal cord injury, we suggest that bladder dysfunction arises from the MS-related spinal lesions. A possible reason why in the MS patients we studied bladder filling exerts no inhibitory effect on somatic spinal motoneurons is that MS-related lesions in the spinal cord remove descending modulation relayed by reticulospinal pathways [19] on propriospinal interneurons. Demyelination due to the spinal lesions, and therefore temporal dispersion in the descending signaling to propriospinal interneurons, alters bladder filling-induced modulation on somatic motoneurons, as happens in patients with spinal transection due to traumatic injuries. Our findings are in line with MRI studies showing that bladder symptoms correlate more with cervical lesions than with suprapontine lesions [20,21]. In this study, we therefore demonstrate with neurophysiological techniques that in patients with MS, despite the prominent suprapontine lesion load, OAB symptoms mainly reflect spinal cord dysfunction. Testing MS patients with both suprapontine and spinal lesions, but no bladder symptoms, would definitively confirm our conclusions. Performing an invasive test, as the urodynamic procedure is, in patients with no bladder dysfunction would, however, raise ethical questions.

Several authors reported a significant improvement of bladder symptoms in MS patients after intradetrusor injections of botulinum toxin, demonstrating the efficacy of various types of botulinum toxin for bladder dysfunction in patients with MS [22,23,24,25,26,27,28,29,30]. Different mechanisms of action have been proposed to explain the long-lasting effects of intradetrusorial botulinum toxin injections, including an effect of botulinum toxin on the afferent, as well as the efferent pathways [6,11]. Investigating the effects of intradetrusorial BoNT/A injection on the H reflex during bladder filling, we found that BoNT/A did not restore the normal H reflex modulation at MCC. Since volumes for first, normal and strong desire significantly increased and detrusor overactivity disappeared after BoNT/A injection, we exclude the possibility that the H reflex modulation was not normalized due to BoNT/A inefficacy on bladder afferent function. Differently from patients with complete spinal lesion due to traumatic injury in whom bladder filling sensation is lost, in the MS patients we studied, we were able to disclose that BoNT/A significantly modulated bladder afferent activity, as shown by the increase in volumes for first, normal and strong desire to void. Since in MS, BoNT/A influences bladder filling sensation, but leaves urodynamic variables testing muscle strength unchanged, we conclude that BoNT/A, at the low dosages we used, prominently modulates bladder afferent information [6,11]. In our previous study in patients with complete traumatic spinal cord lesion [6], we observed that, unlike healthy subjects, H reflex increases at maximum bladder filling. BoNT/A-induced reduction of bladder afferent inflow to spinal cord turned the H reflex facilitation toward a slight decrease of H reflex size at MCC, thus making the BoNT/A effects apparent. The MS patients we studied, who did not have a complete spinal cord injury, showed a reduced decrease in H reflex size instead of an increased H reflex size at maximum bladder filling before BoNT/A. We therefore believe that in our MS patients, BoNT/A-induced effects bladder afferent input, and therefore, its effects on the H reflex modulation during bladder filling are unappreciable.

## 5. Conclusions

Our study with H reflex testing during bladder filling shows that in patients with MS having both suprapontine and spinal disease-related lesions, neurogenic overactive bladder is more likely due to spinal than to suprapontine lesions. BoNT/A-induced improvement in bladder dysfunction relies on changes in bladder afferent signaling, as shown by the increase in volumes for first, normal and strong desire.
